# Five point initiative: a community-informed bundled implementation strategy to address HIV in Black communities

**DOI:** 10.1186/s12889-023-16525-7

**Published:** 2023-08-25

**Authors:** Sannisha K. Dale, Kayla Etienne, Sidny Hall, Kimberly Lazarus, Kalenthia Nunnally, George Gibson, Roxana Bolden, Nadine Gardner, Jasmyn Sanders, Rachelle Reid, Arnetta Phillips

**Affiliations:** 1 Department of Psychology, University of Miami, 5665 Ponce de Leon Blvd, Miami, FL 33146 USA; 2 New College of Florida, Sarasota, FL USA; 3 Blessing Hands Outreach, Miami, FL USA; 4 Flashlight of Hope, Miami, FL USA; 5 A Sister with A Testimony, Miami, FL USA

**Keywords:** HIV, Implementation, Community-Based Participatory Research, Black Communities, Five Point Initiative

## Abstract

**Background:**

Black individuals in the U.S. remain the most disproportionately impacted by new HIV diagnoses, represent the highest portion of individuals living with HIV, and have the highest morbidity rates. Structural inequities and historical oppression are the primary drivers. Such drivers limit access to HIV prevention tools that need to be delivered with culturally congruent and community-informed approaches.

**Methods:**

The Five Point Initiative (FPI) is a community-informed bundled implementation strategy developed and piloted between September 2019 and March 2020 in Miami, Florida in communities heavily impacted by HIV. Key components of the strategy included community consultants/experts, five categories (hence the “Five Point”) of community businesses (e.g., corner stores, beauty supply stores, laundromats, mechanics, barbershops), local health organizations, an academic research program engrossed in community engaged research, and community residents who provided ongoing feedback throughout. Outcomes of FPI included (a) survey information (e.g., knowledge of and access to PrEP, barriers to care) and pilot data (acceptability and feasibility), (b) expansion of reach to Black individuals in HIV high impact zip codes in Miami, (c) insights on our bundled implementation strategy, (d) condom distribution, and (e) HIV testing.

**Results:**

Over the course of six months FPI carried out 10 outreach events, partnered with 13 community businesses and 5 health organizations, engaged 677 community residents, collected health information via a survey, distributed 12,434 condoms, provided information on PrEP, and offered voluntary HIV testing (131 completed). FPI’s ability to reach residents who are not being reached (e.g., 68.8% never heard of PrEP, 8% no HIV testing ever, 65.9% no primary care provider), positive feedback from residents (e.g., 70% very satisfied, 21% satisfied; 62% strongly agree and 25% agree they would participate again) and qualitative interviews with businesses provide evidence of acceptability and feasibility. Further, survey data provided insights on factors such as socio-demographics, discrimination experiences, barriers to care, social-structural factors, physical and sexual health, and mental health and substance use.

**Conclusions:**

The FPI bundled implementation strategy shows promise to deliver health prevention/intervention for HIV and other health conditions to communities facing health inequities and for whom the current system for delivering care is insufficient.

**Supplementary Information:**

The online version contains supplementary material available at 10.1186/s12889-023-16525-7.

## Introduction

Black individuals remain the most disproportionately impacted racial group by HIV in the United States. In 2019, Black persons represented 42% of individuals newly diagnosed with HIV and in 2018 41% of individuals living with HIV [1]. This disparity is linked to structural factors including racism, heterosexism, poverty, HIV stigma, harmful laws/policies, lack of access to adequate and culturally competent physical/mental health care, trauma/violence, unaddressed mental health struggles, and underutilization of community-based approaches to offset some barriers to access [2–9]. These issues are evident in Miami, FL which continues to rank # 1 among U.S. cities in terms of new HIV diagnoses and suboptimal outcomes along the treatment cascade (e.g., number of people living with HIV engaged in care, retained in care, and who has HIV viral suppression) [10]. While Black individuals make up 16% of the population in Miami they account for 30% of new HIV diagnoses with 1 in 31 Black individuals living with HIV in Miami [1]. There is a core prerequisite that is needed in order to fulfill the four pillars (diagnose, treat, prevent, and respond) of the Ending the HIV Epidemic (EHE) initiative – that is the ability to reach and engage. HIV health equity scholars challenge the notion that Black individuals placed at risk for living with HIV are “hard to reach” and call for successful approaches in reaching Black communities [11–13]. One approach has been partnering with venues frequently accessed within Black communities [14–17]. For instance, health departments have partnered with barbershops and hair salons [18] and research supports the feasibility of conducting intervention studies there [14]. Similarly, churches have shown promise in increasing HIV testing levels [11, 18–20]. However churches sometimes struggle to (a) promote sexual health within the confines of their views on sex and sexuality [21] and (b) engage younger generations and those marginalized by heterosexism and cisgenderism [22].

Going beyond churches, public parks, homeless shelters, and bars with mobile HIV testing sites have caused an increase in HIV testing, many of which were people who had never previously tested [20, 23, 24]. Venue-based testing also provides rapid results and more guaranteed receipt of results  [25, 26]. Further, rapid testing used at community sites detect and diagnose HIV at an earlier stage than non-rapid tests many clinics/health centers use  [27]. Increasing knowledge about pre-exposure prophylaxis (PrEP) and access to PrEP via venues also has the potential to lower rates of HIV transmission. Black individuals are less likely to know about PrEP, have discussed PrEP with a provider, or utilized PrEP [28–30]. However, recent mobile efforts to encourage PrEP usage and continuation have increased PrEP adherence [19].

For community and venue-based efforts we can leverage data from departments of public health to focus on highly impacted zip codes. Dynamic HIV transmission maps also suggest areas in need of HIV outreach programs [31]. Lastly, local expertise can identify venues impacted by factors linked to HIV transmission (e.g. substance use, sex work, homelessness) [32]. In addition, outreach efforts by community-based organizations tend to succeed with goals of increasing HIV testing, education, and treatment [23, 33, 34]. For academic and community partnerships the use of community-based participatory research (CBPR) is central in identifying needs, locally relevant strategies, and promoting HIV testing and education [35–37].

Given the necessity to address HIV health inequities faced by Black individuals in Miami, FL, innovative strategies that build on existing literature and harness local resources and community partnerships are needed. As such, the Five Point Initiative (FPI) was developed in close collaboration with community experts and piloted to assess preliminary acceptability and feasibility and to improve education and knowledge about HIV prevention and treatment, access to HIV testing, PrEP information, and condom usage.

## Methods

### Overview of the Five Point Initiative Model

**Fig. 1:**
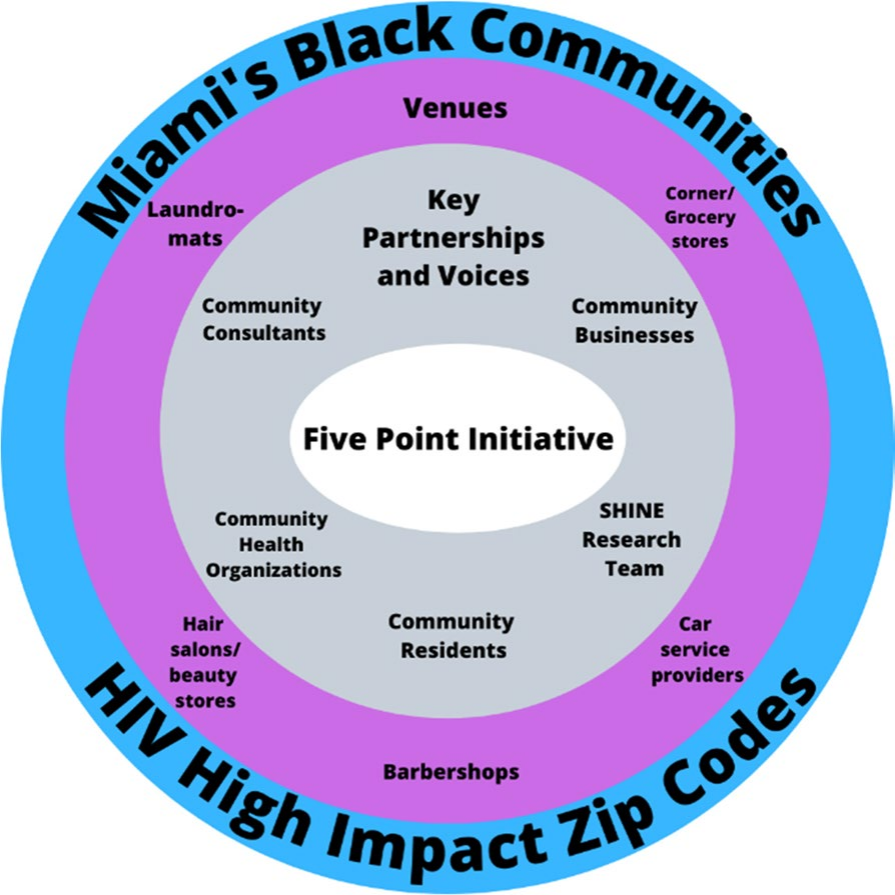
Five point initiative components and context

The Five Point Initiative pilot (1) partnered with five categories of businesses that Black individuals may frequent (i.e., corner/grocery stores, laundromats, salon and beauty supply stores, barbershops, and car service providers) in Miami Dade zip codes with the highest number of Black individuals living with HIV (2) closely collaborated with community health organizations funded by the Centers for Disease Control and Prevention (CDC), Health Resources and Services Administration (HRSA), and/or Substance Abuse and Mental Health Services Administration (SAMHSA) and (3) hosted outreach events in which community members complete a brief electronic survey in exchange for a service/voucher (e.g. free laundry wash and dry) at a venue with the cost being covered for by research funding and are offered HIV/STI voluntary counseling and testing on a mobile health unit, PrEP information, and condoms. Outcomes of the Five Point Initiative included (a) survey information (e.g. knowledge of and access to PrEP, barriers to care) and pilot data (acceptability and feasibility), (b) reach of Black individuals in HIV high impact zip codes in Miami who are not being reached by traditional approaches in terms of HIV prevention/treatment efforts as evidenced by lack of knowledge of PrEP and HIV testing, (c) insights from residents and business partners on our local implementation strategy, (d) condom distribution, and (e) HIV testing. As depicted (see Fig. 1), there were five key partnerships/voices and five categories of businesses with efforts targeting HIV high impact zip codes for Black individuals in Miami, FL.

### Community consultants

Community consultants were central to the Five Point Initiative model and bring decades of expertise. One consultant had over twenty years of experience working in Black communities on HIV prevention and treatment, LGBTQ+ advocacy, harm reduction for substance use, increasing access to resources, grief counseling, and operating a faith-based ministry. The second consultant had over twenty years of experience establishing/directing nonprofits to advocate for women and girls' sexual health and mobilizing Black women in the fight against HIV. These consultants provided key insights for planning, executing, and improving FPI, recruited businesses/venues for partnerships, and assisted with outreach events. To recruit businesses falling within the categories noted below, the consultants approached businesses (many of which they had pre-existing connections with) located in the identified HIV high impact zip codes that serve and attract Black residents and discussed FPI. Having an intimate understanding of the businesses and locations consultants also helped to inform the potential days/times for the events to maximize resident engagement.

### Community businesses

Through conversations upon initial recruitment, ongoing dialogue to plan an outreach event, and exit interviews, community businesses provided insights to make the events successful and improve the approach. The five types of businesses were corner/grocery stores, laundromats, salon and beauty supply stores, barbershops, and car service providers (e.g., car wash, gas station, mechanic). Businesses distributed flyers to patrons weeks leading up to the event. The $20 vouchers provided to participants were used to purchase products or services from the businesses and generated revenue. Based on interest, neighboring businesses were simultaneously partnered with during one event.

### Community health organizations

To work collectively with organizations with a shared mission to address HIV, FPI partnered with community health organizations (CHO) including clinics, local HIV prevention centers, and the department of health to provide mobile HIV testing and PrEP screening and referrals when indicated.

### Residents

All community residents (18 years and older) were invited to participate during outreach events as they visited the businesses or passed nearby. Consent was obtained verbally (approved by University of Miami Institutional Review Board) and description of the research study and requirements were provided in written form and orally to participants. Residents who chose to participate completed a survey, were offered HIV testing, information on PrEP, and condoms, and received a business voucher. Residents provided satisfaction ratings, feedback, and insights as they participated and via questions at the end of the survey.

### Strengthening Health through Innovation and Engagement (SHINE) research program

The research program carriers out a suite of projects (a) addressing inequities at the intersection of HIV and mental health especially among individuals minoritized due to racism (e.g., Blacks/African Americans), sexism, heterosexism, and cisgenderism and (b) engaging community members and stakeholders in research. The research team, reflective of the racial/ethnic communities most impacted by HIV, consists of the principal investigator/director, research staff, postdoctoral and doctoral students in psychology and public health, and undergraduate students. Research staff played a key role in coordinating the event logistics, all team members assisted with participant engagement at events, and oversight was provided by the principal investigator. In addition, a weekly meeting and debrief was conducted with the research team and community consultants.

### HIV High-Impact Zip Codes

The piloting of FPI focused on five zip codes and Black communities in Miami, FL where HIV prevalence is high. The goal was to conduct an event at each of the five types of venues within these zip codes.

### Survey administered to residents via RedCAP [38]

Residents were given the option to complete the survey on their own (using smartphone or a tablet/iPad issued by the team) or have it read by a team member. The survey was available in English, Spanish, and Haitian Creole and captured information on demographics, life experiences, mental health, substance use, physical health, sexual health, and event feedback.

### Socio-demographics

Twelve questions asked about participant age, birth country, work status, household income, educational level, housing, gender identity, sex assigned at birth, relationship status, sexual orientation, racial identity, and ethnic identity.

### HIV status

Participants were asked to select one of the following regarding HIV status: HIV-positive detectable viral load, HIV-positive undetectable viral load, HIV-positive I don't know my viral load, HIV-negative, or I don't know.

### Housing stability/food insecurity

Two items were used from the United States Department of Agriculture’s 18-item scale [39] to determine food insecurity and hunger (e.g., “In the past 12 months, the food I bought just didn’t last and I didn’t have money to get more”).

### Child care needs

From a national study on affordable childcare [40] we used two items (e.g., “How serious of a problem is finding quality, affordable childcare in your area?”).

### Overall health

Participants were asked to rate their own health choosing from: poor, fair, good, very good, or excellent. In addition, participants were asked if they had a primary care doctor.

### Mental health

From the Patient Health Questionnaire (PHQ-9) [41, 42], we used four items to assess the participant's mood from the past two weeks (e.g. feeling down, depressed, or hopeless). In addition, participants were asked one question on self-esteem (i.e., I have high self-esteem) and one on trauma) [43].

### Substance use

Two questions asked about alcohol use and drug use: “How many times in the past year have you had four or more drinks in a day?” and “How many times in the past year have you used a drug or used a prescription medication for non-medical reasons?”

### Sexual health and health behaviors

Participants were asked 11 questions (varied based on HIV status and branching logic) about sexual health and health behaviors. Questions were: When was the last time you were tested for HIV? Have you ever spoken to a doctor about HIV? Have you had any sex without a condom in the past 3-months? Are you currently prescribed HIV medication?, In the last 4 weeks, how good a job did you do at taking your HIV medicine in the way you were supposed to? Have you ever heard of PrEP?, Have you ever spoken to a healthcare provider about getting PrEP?, Are you currently prescribed PrEP?, On a scale from 0-10, how important is it to you to start PrEP?, On a scale from 0-10, how confident are you that you will start using PrEP?, [30] and In the last 4 weeks, how good a job did you do at taking [PrEP] in the way you were supposed to?

### Everyday discrimination

Five items were used from the Everyday Discrimination Scale  [44] ( e.g., “You are treated with less courtesy or respect than other people”, “you are threatened or harassed”), which asks participants to note in their day-to-day life whether they have experienced discrimination, how frequently (e.g., almost every day, at least once a week), and to indicate the identity that was targeted (e.g., race, gender).

### Barriers to medical and mental healthcare

Eight items were adapted from Heckman’s scale on barriers to care among people living with HIV [45] and assessed the following barriers for people living with and without HIV: financial reasons, HIV stigma, lack of transportation, housing, language spoken, competency of providers, shortage of mental health providers, and distance to the facilities.

### Medical mistrust

Five items by LaVeist [46] on mistrust for medical facilities and personnel were used (e.g., “Hospitals have sometimes done harmful experiments on patients without their knowledge”).

### Community evaluation feedback

Eight questions captured resident’s satisfaction, where residents heard about the event, activities they engaged in (e.g., testing), whether they would participate in another activity, what aspects should be in future events (e.g., voucher), and their thoughts about the event.

### Exit interview with businesses

Manager/owners were asked five questions on satisfaction with the event and planning, interest in collaborating in future events, areas for improvement, and overall comments.

### Statistical analyses

SPSS version 28 was used to perform all statistical analyses. All 654 participants who completed surveys were included in the quantitative analyses. Descriptive statistics (e.g., mean, standard deviation, frequencies) were computed for all quantitative variables. The brief semi-structured interviews with business partners were reviewed for common themes by two team members under the guidance of the PI.

## Results

Between September 2019 and March 2020 (paused due to COVID-19) in four HIV high impact zip codes in Miami, FL, 10 outreach events were conducted in collaboration with 13 businesses and 5 health organizations. In total 677 residents were engaged, 654 people completed surveys, 131 volunteered for HIV testing, and 12,434 condoms were distributed. We partnered with 4 corner/food stores, 3 barbershops, 2 beauty supply stores/ hair salons, 1 laundromat, and 3 businesses (1 feminine health and 2 clothing/accessories) that are categorized as “Other”. In general, the corner/food store events had the highest average for residents who completed surveys and testing per event (averaged 93 surveys and 18 tests across 4 events), followed by the laundromat (averaged 49 surveys and 19 tests for 1 event), barber/salon/beauty (averaged 42 surveys and 9 tests across 3 events), and the other category (averaged 22 surveys and 2 tests).

### Socio-demographics of residents

Among participants 74.1% were born in the United States, with 93.1% speaking English as their primary language (see Table 1). Median age was 42 years old, 53.7% identified as female, 42.4 % as male, 0.2% transgender, and 0.6% percent as gender non-conforming. The majority (80.7%) identified as heterosexual and 12.3% identified as LGBQ+. Participants largely identified as Black/African American (84.1%) with 35% being Afro-Caribbean Black (non-Haitian) and 13% Haitian/Haitian American. Household income was less than $5000 a year for 22% and 41.9% were working full time.

**Table 1:** Demographics

**Demographics**	**Frequency (/mean)**	**Percent (/SD)**
Age (average)	42.42	15.47
Born in the US	495	74.1
Languages spoken		
English	622	93.1
Kreyol (Haitian Creole)	61	9.1
Español (Spanish)	68	10.2
Other	12	1.8
Gender identity		
Male	283	42.4
Female	359	53.7
Trans male/ trans man	1	0.1
Trans female/ trans woman	1	0.1
Genderqueer/ gender non-conforming/non-binary	4	0.6
Different identity	0	0
Sex assigned at birth		
Male	281	42.1
Female	363	54.3
Intersex	4	0.6
Sexual orientation		
Heterosexual	539	80.7
Gay	11	1.6
Lesbian	13	1.9
Bisexual	25	3.7
Queer	5	0.7
Pansexual	3	0.4
Asexual	12	1.8
Unsure/ questioning/ exploring	17	2.5
Not listed	23	3.4
Relationship status		
Married	51	7.6
Not married, but living with someone as if married	22	3.3
Non- cohabiting relationship	13	1.9
Single	11	1.6
Divorced or separated	169	25.3
Loss of long-term partner/ widowed	14	2.1
Choose not to answer	12	1.8
Racial ethnicity		
Black/ African American	562	84.1
Asian	3	0.4
White (including White Hispanic /Latinx)	57	8.5
Native Hawaiian or other Pacific Islander	3	0.4
Native American	3	0.4
Multi-Racial/ mixed	4	0.6
Different racial identity	16	2.4
Ethnicity		
Haitian American	87	13
Afro-Caribbean Black (not Haitian)	234	35
Hispanic or Latino	67	10
Not Hispanic, Latino, Haitian, or Afro- Caribbean	271	40.6
Highest or current education level		
Eighth grade or lower	27	4
Some high school	104	15.6
High school graduate or GED	225	33.7
Some college	143	21.4
College graduate	86	12.9
Some graduate school	18	2.7
Graduate school degree	37	5.5
I choose not to answer	8	1.2
Current housing arrangement		
Renting home or apartment	359	53.7
Living in home or apartment owned by you or someone else in the household	172	25.7
Residential drug, alcohol, or other treatment facility	9	1.3
Publicly subsidized housing (like section 8)	25	3.7
A friend or relative’s home/ apartment	45	6.7
Temporary/ transitional housing (e.g., hotel, AIDS specific housing, sober living)	5	0.7
Homeless: sleeping in a shelter	10	1.5
Homeless: sleeping on the street, beach, car etc.	5	0.7
Other	3	0.4
I choose not to answer	15	2.2
Total household income, before taxes and other deductions, during the past 12 months		
Less than $5,000	147	22
$5,000 through $11,999	99	14.8
$12,000 through $15,999	55	8.2
$16,000 through $24,999	71	10.6
$25,000 through $34,999	46	6.9
$35,000 through $ 49,999	62	9.3
$50,000 through and greater	62	9.3
Don’t know	64	9.6
Refuse to answer	42	6.3
Work		
Full-time	280	41.9
Part-time	133	19.9
Full-time or part-time in school	27	4
Neither in work nor in school	80	12
On disability	86	12.9
Other	48	7.2
I choose not to answer	35	5.2

### Discrimination, barriers to care, and other social-structural factors

A high percentage of participants reported experiencing discrimination almost every day: 50.4% receive poorer service than other people at restaurants, 65.1% people act as if they are afraid of you, 71.4% are threatened or harassed (see Table 2) with various identities as the target of discrimination (gender 48.4%; gender identity 9.7%; race/ethnicity 78.8%; sexual orientation 4.7%; living with HIV 6.7%). Participants endorsed moderate levels of medical mistrust (avg = 3.13). Residents also reported barriers to accessing healthcare services they need: 18.9% long distances 23.6% transportation, 20.4% providers who do not speak their language, 38.3% financial resources, 35.6% lack of affordable housing, and 36.9% stigma against persons living with HIV. Food security was an issue with 40.7% reporting that “often” food bought did not last and they had no money to get more. Participants reported an average of 2.33 children and 21.8% found it somewhat difficult or very difficult to find affordable childcare. Lastly, 26.8% reported incarceration history.

**Table 2:** Discrimination, barriers to care and other social-structural factors

	**Frequency (/mean)**	**Percent (/SD)**
Medical mistrust (average)	3.13	1.03
Everyday discrimination		
Treated with less courtesy or respect than other people		
Almost everyday	283	42.4
At least once a week	73	10.9
A few times a month	91	13.6
A few times a year	50	7.5
Less than once a year	72	10.8
Never	78	11.7
Receive poorer service than other people at restaurants		
Almost everyday	337	50.4
At least once a week	95	14.2
A few times a month	103	15.4
A few times a year	52	7.8
Less than once a year	33	4.9
Never	27	4.0
People act as if they think you are not smart.		
Almost everyday	332	49.7
At least once a week	77	11.5
A few times a month	82	12.3
A few times a year	59	8.8
Less than once a year	40	6.0
Never	57	8.5
People act as if they are afraid of you		
Almost everyday	435	65.1
At least once a week	46	6.9
A few times a month	60	9.0
A few times a year	36	5.4
Less than once a year	29	4.3
Never	41	6.1
You are threatened or harassed.		
Almost everyday	477	71.4
At least once a week	70	10.5
A few times a month	38	5.7
A few times a year	21	3.1
Less than once a year	14	2.1
Never	27	4.0
Identity targeted by** e**veryday discrimination^**a**^		
Gender	323	48.4
Gender identity	66	9.7
Race or ethnicity (Black, Latinx, etc.)	526	78.8
Living with HIV	46	6.7
Your sexual orientation (LGBTQ)	32	4.7
Other	166	24.8
Barriers to care		
Long distances to medical facilities and personnel	122	18.3
Lack of transportation	153	22.9
Providers who do not speak your language	132	19.8
Lack of health care professional who are adequately trained and knowledgeable	150	22.5
Shortage of psychologists, social workers, and mental health counsellors to help address mental health issues	162	243
Personal financial resources	246	36.8
Lack of adequate and affordable housing	238	35.6
Community residents’ stigma against persons living with HIV/ AIDS	142	21.3
Incarcerated	179	26.8
Time in jail	139	20.8
Time in prison	48	7.2
Never	116	17.4
Food bought didn’t last and you had no money to get more		
Often	272	40.7
Sometimes	266	39.8
Never	109	16.3
Overall food insecurity (average)	1.47	1.37
Number of children.	2.33	3.09
Finding quality, affordable childcare that’s convenient for your family		
Very easy	130	19.5
Somewhat easy	145	21.7
Somewhat difficult	73	10.9
Very difficult	73	10.9
I haven’t needed childcare	52	7.8
How serious of a problem is finding quality, affordable childcare in your area?		
Very easy	140	21
Somewhat easy	100	15
Somewhat difficult	74	11.1
Very difficult	87	13
I haven’t needed childcare	68	10.2
Overall childcare (average)	3.17	2.54

For barriers to care (e.g., Community residents’ stigma against persons living with HIV/ AIDS) participants indicated with a “yes” or “no” response “which of the following have made it difficult for you to receive the healthcare services you need.”

^a^number indicates percent of the time that the noted identity was targeted by everyday discrimination. For barriers to care (e.g., Community residents’ stigma against persons

### Physical and sexual health

In rating their general health, 35.8% selected “good” and 24.8% rated poor/fair (see Table 3). Among participants 65.90% did not have a primary care physician. Regarding HIV status and testing, 5.1% reported living with HIV and adhering to their HIV medication (.06 % poor, 22% fair, .06 % good, .17 % very good, 50% excellent), 8.4% did not know their HIV status, 17.1% had been tested for HIV over 1 year ago, and 8.8% had never been tested for HIV in their lifetime. For condom usage, 21.6% of participants had sex without a condom one or two times in the past 3 months. In regards to PrEP, 66.8% of participants had never heard of PrEP before and both their views on how important it was to start PrEP (avg = 3.37) and their confidence in starting PrEP were low (avg = 2.59).

**Table 3:** Physical and sexual health

	**Frequency (/mean)**	**Percent (/SD)**
General health		
Poor	21	3.1
Fair	145	21.7
Good	239	35.8
Very good	142	15
Excellent	100	15
Have a primary care doctor	439	65.7
HIV status		
HIV-positive, detectable viral load	9	1.3
HIV-positive, undetectable viral load	19	2.8
HIV-positive, I don’t know my viral load	7	1
HIV-negative	556	83.2
Don’t know	56	8.4
Last time tested for HIV		
In the last 3 months	238	35.6
3 to 6 months ago	121	18.1
6 to 12 months ago	68	10.2
Over 1 year ago	114	17.1
I can’t remember when	44	6.6
I have never been tested for HIV	59	8.8
Spoken to a doctor about HIV	404	60.5
Sex without a condom in the past 3 months		
Yes, 1 or 2 times	144	21.6
Yes, 3 or 5 times	53	7.9
Yes, more than 5 times	68	10.2
No	379	56.7
Knowledge of PrEP		
Yes	201	30.1
No	446	66.8
Spoken to provider about PreP	87	13.0
Currently prescribed anti-retrovirals^a^	18	51.4
Currently prescribed PrEP	24	3.6
Importance in starting PrEP (average)	3.37	3.92
Confidence in starting PrEP (average)	2.59	3.56
ART adherence (average)^b^	3.83	1.43
PrEP adherence (average)^c^	2.96	1.57

^a^Number and percentage were out of the number of residents who are people living with HIV

^b^Average is among PLWH who are currently prescribed anti-retrovirals

^c^Average is among people who are currently prescribed PrEP

### Mental health and substance use

Trauma exposure was high with 39.1% having witnessed, experienced, or dealt with a traumatic event in their lifetime (see Table 4). In the past two weeks many participants reported several days or more of feeling anxious (37%) and depressed/hopeless (35.7%). Substance use varied with most participants reporting never having 4 or more alcoholic drinks in one day (52.4%) or using drugs in the past year (79.9%). However, when asked on a scale ranging from 1 (not very true of me) to 5 (very true of me) about self-esteem participants reported high self-esteem (avg = 3.97).

**Table 4:** Mental health and substance use

	Frequency (/mean)	Percent (/SD)
Feeling nervous, anxious or on the edge		
Not at all	400	59.9
Several days	151	22.6
More than half the days	44	6.6
Nearly everyday	52	7.8
Not being able to stop or control worrying		
Not at all	410	61.4
Several days	138	20.7
More than half the days	52	7.8
Nearly everyday	47	7
Little interest or pleasure in doing things		
Not at all	403	60.3
Several days	136	20.4
More than half the days	61	9.1
Nearly everyday	44	6.6
Feeling down, depressed, or hopeless		
Not at all	406	60.8
Several days	156	23.4
More than half the days	44	6.6
Nearly everyday	38	5.7
I have high self-esteem (average)	3.97	1.36
Experienced, witnessed, or dealt with any traumatic event	261	39.1
Overall anxiety (average)	2.23	1.68
Overall depression (average)	2.18	1.55
Overall depression and anxiety (average)	4.40	2.98
Alcohol consumption (>/= 4 drinks in a day) in the past year		
Never	350	52.4
Less than a month	111	16.6
A few times per month	82	12.3
Few times a week	51	7.6
More than 3x a week	50	7.5
Substance use		
Never	534	79.9
Less than a month	31	4.6
A few times per month	19	2.8
Few times a week	26	3.9
More than 3x a week	34	5.1
Type of drug use		
Marijuana use	77	11.5
Stimulant use	16	2.4
Heroin use	5	0.7
Tranquilizer use	1	0.1
Non-medical use of prescription medications	18	2.7
Club drug use	8	1.2
Other	6	0.9
Overall alcohol/substance use (average)	0.70	1.00

### Evaluation of community event

Participants were very satisfied with the FPI events and indicated that they would participate in future events (see Table 5). For instance, 70% were very satisfied, 21% were satisfied, 62% strongly agreed, and 25% agreed they would participate again. Participants also indicated aspects of the event that should be included in the future, time they would be willing to spend engaging at the business location, and activities they would be willing to do in this location in exchange for a voucher (e.g., 60% would get tested for HIV/STI, 44% would get a prescription for HIV prevention/treatment). Participants’ responses to an open-ended question about their thoughts of the event were also overwhelmingly positive with responses like, “It's good because people are trying to help the community and most people don't care about the hood, and it's showing that people can do good” and “I feel like this is very helpful and informative. The free HIV testing is very helpful along with the information and the statistics that was provided to me. The free wash is also helpful for those that aren't financially stable and able to wash their clothes. I would definitely participate in the next event.” Most participants heard about the event through friends or family (36.2%).

**Table 5:** Residents evaluation of community event

	Frequency (/mean)	Percent (/SD)
Heard about our event via:		
Friend or family	233	36.2
Word of mouth	105	15.7
Flyer	59	8.8
Event venue	247	37
Activities taken part in during today’s event.		
Completed survey	593	88.8
Free HIV testing	92	13.8
Free condoms	176	26.3
Satisfaction with the event		
Very satisfied	461	70.4
Satisfied	137	20.9
Neither satisfied nor unsatisfied	30	4.6
Unsatisfied	9	1.4
Very unsatisfied	14	2.1
Would participate in another event		
Strongly agree	409	62.4
Agree	165	25.2
Neutral	52	7.9
Disagree	11	1.7
Strongly Disagree	14	2.1
Aspects that should be included in future events.		
Tablets to conduct surveys	391	58.5
Vouchers	365	54.6
Host the event at similar locations (hairdressers, barbershops, corner stores, laundromats, mechanics)	280	41.9
Time willing to spend doing study/ intervention in this location.		
Less than 15 minutes	271	40.6
15-30 minutes	215	32.2
30-45 minutes	85	12.7
45 minutes or above	73	10.9
Research study activities willing to do in this location in exchange for a voucher.		
Talk to someone 1 on 1	468	70.1
Watch videos	419	62.7
Complete survey on tablets	570	85.3
Watch a speaker give a group presentation	425	63.6
Get tested for HIV and other STIs	403	60.3
Get a prescription for medication to prevent HIV or treat HIV	293	43.9

### Exit interview with business

All thirteen businesses completed the exit interviews and seven common themes were identified (see Table 6): (1) organization and planning, (2) increase in daily revenue and customers (3) increase in HIV awareness and education, (4) interest in future collaboration, (5) satisfaction with the team, (6) improvements, and (7) community enrichment. Six out of the thirteen business owners/managers mentioned an increase in daily revenue and customers served and they commended the team’s positive interactions with their customers. Some owners noted that events like these are rare (e.g., “You know nobody ever did. I mean unless you go to a doctor.”). In addition, all business owners were interested in collaborating in the future with eleven of the participants replying “definitely” and one replying “absolutely”.

**Table 6:** Illustrative quotes from exit interview with businesses

**Categories**	**Illustrative Quotes**
Organization and Planning	“So, it’s like you had everything, you had your banister, your advertisement. You know? It was definitely enough parking, enough room for everyone to be seated. Sometimes it’s in smaller spaces, but you know the space was a nice size. I think everything was very professional” “The planning process was pretty… it was pretty good. I would say out of 1 out of 10, I give it a 10.” “It was organized. It was cool because you guys did the vouchers. When I went to get like the products from the store, I was like oh wow!”
Increase in daily revenue and customers	“Yeah we saw some customers that we never saw here.” “Yeah. I saw new customers.” “Very satisfied! I didn’t think that many people would come and at least 100 people came and did the testing” “I would like to say the fact that the customers did come to the store. It was like wow. This really works.”
Increase in HIV awareness and education	“Raising awareness. It’s a good thing among people. Checking if they have HIV, providing condoms and everything. So, it’s something necessary I guess for the community” “Very satisfied because the people were happy. I mean someone came out on the streets and talked to them about certain stuff. You know nobody never did. I mean unless you go to a doctor.” “They need the education. A lot of young people drop out from school and stuff and they’re out there on the streets, so they need their little guidance. You know? They don’t know. So, a little education will help them. You know? And talk about life because that’s what they need.”
Interest in future collaboration	“Definitely.” “Definitely… Because awareness. To keep awareness” “Definitely. Because I like helping people. I love putting smiles on other people’s faces and that is what you guys are into and so I would love to be a part of it all. At all times.”
Satisfaction with Shine Team	“I have a lot of compliments from people who have dealt with you…. They said ya’ll was nice.” “I just want to thank you… you know choosing me for your guy’s event… Like I said, it looks like everybody had a nice time. Everything went smoothly from what I can see. “ “Very satisfied. Because I love the way you guys interact with all the people” “I loved everything about the event.” “I wish there were more people like you guys to come and talk to them one on one… I was happy you guys came out and did that”
Improvements	“Everything went good, but the only thing is it did need more time to let the people know. It was so sudden. It was just one week advertising. It should be advertised in a month before I go in with it. You know? Probably next time would be greater… It just needs more time to advertise. You guys need to do one more. I would tell you guys to advertise it for at least a month before we go ahead.” “Probably two weeks to twenty days prior and then three different phases. Promote it one week, then promote it the second week a little bit harder, then the final couple days promote it the max it could be done.” “Maybe planning ahead of time. Maybe a couple of months before so we can tell the people and go advertise it.”
Community Enrichment	“It helps up the community. It gets to know people around and it really helps out a little. I mean there are times I go out there and you know you, the community is doing a health incentive to get out there and promote it and make it happen.” “Keep doing a great job. Keep up the good work because those people really need you…. A lot of them don’t have families, you know don’t do stuff and don’t give them anything for a holiday, their birthday, or anything else. They really appreciated it because I talked to them like, to make them feel loved and wanted and you know? You have something. So, all around it was a great event.” “I see some results. They came in especially the young people. That is what young people need.”

## Discussion

The Five Point Initiative is an innovative bundled implementation strategy for reaching and engaging Black communities around sexual health and HIV testing and prevention. FPI centers the expertise of community consultants with in-depth knowledge of the local communities and venues, partners with community venues that are frequented by Black residents in a manner that not only asks for altruism but increases their daily revenue, leverages the resources and partnerships with community health organizations, listens throughout interactions with residents and businesses for ways to improve, and is carried out with the support and coordination of an academic partner and research program that centers equity and is committed to addressing HIV inequities. In piloting FPI important data was gathered that supports feasibility and acceptability and will help us to better understand the interplay between neighborhoods, social-structural factors, physical and sexual health, and mental health. However, perhaps the most valued aspect by residents and partners was not the data, but the services, information, and resources delivered during the outreach events.

Within six months almost 700 residents were engaged and the success is consistent with literature indicating that venue-based approaches, using CBPR elements, done in collaboration with local businesses and health organizations (/mobile units), and leveraging local expertise can be successful in expanding the reach of HIV prevention efforts to minoritized communities [23, 33–37, 47, 48]. Further, the feedback from both residents and community businesses were positive with responses indicating that this approach to health promotion (coming to the venues, providing a voucher, team that racially/ethnically reflects the communities) is something they appreciated and had not seen before. As a result, all businesses noted that they would partner again for FPI and the majority of residents would participate again. All residents were educated about PrEP, provided condoms, and offered voluntary HIV testing; however, only about one fifth of residents volunteered to get tested for HIV suggesting that requiring HIV testing for the voucher in FPI (present in ongoing work) would lead to a higher number of residents testing for HIV.

Survey data on sociodemographics, discrimination, barriers to care, other social-structural elements, physical and sexual health, and mental health and substance use highlighted experiences and issues being faced by residents. Socio-demographic findings indicate that FPI is able to reach diverse Black residents for HIV prevention efforts. A significant percent of residents reported facing everyday discrimination (including threats and harassment) with various identities being targeted (race/ethnicity, gender identity, sexual orientation, and HIV status) [2–4]. Many reported barriers to care (e.g. transportation, finance, housing, and stigma), moderate levels of medical mistrust, and food insecurity. These barriers to care affirm the need to reach residents where they reside to deliver health promotion and care. Also given ongoing racism, the presence of a majority Black team and community consultants (known in the communities) at the outreach may have offset initial hesitations by residents, created a welcoming atmosphere, and enhanced trust. Lastly, the vouchers when partnering with food venues was likely an attractive incentive in the context of food insecurity and food venues had the highest average engagement of residents compared to other businesses partnered with.

In terms of physical and sexual health, survey responses highlighted that the majority of residents did not have a primary care provider, some had never been tested for HIV in their lifetime, a significant portion had condomless sex within the past three months, and most had never heard of PrEP. Our findings reiterate that Black residents are not being adequately reached with HIV biomedical prevention options [49, 50]. Fortunately post survey completion FPI provided information about PrEP and how residents can access it. Findings also indicate that mental health interventions and resources are needed as well as a nuanced understanding of what mental health struggles may be most prevalent.

There are additional implications. First, having paid community consultants from local communities is pivotal in building community partnerships and having lived expertise on the local context. Second, FPI may be used to target other health issues (e.g., screenings, vaccines) and provide immediate access to PrEP. For instance, community health partners on mobile units with appropriate credentials (e.g., nurse practitioner) can screen participants for PrEP and provide same day prescriptions. Third, weekly debriefs among the team (e.g., community consultants, research team), exit interviews with businesses, and formal and casual feedback from residents suggested decreasing the number of survey questions, increasing the number of mobile health units available when a large number of residents are anticipated, and advertising for the event at least a month or more in advance. Fourth, examining community reach and testing trends of the community health organizations partnered with may indicate if FPI participation expands their reach. Further, given that specific geographical locations were targeted, combining census data on neighborhood factors with collected data may provide insights. Lastly, a large-scale cluster randomization research project is needed to assess the effectiveness of the FPI bundled implementation strategy.

In summary, the Five Point Initiative is a promising bundled implementation strategy. Findings provide evidence of feasibility and acceptability with widespread enthusiasm from businesses and residents as well as data highlighting inequities facing primarily Black residents in HIV high impact communities. Gone are the days when major hospitals should be viewed as the home of prevention and care, individuals and especially those who have historically been neglected by such institutions need to be met in their communities and at places they frequent using promising approaches.

### Supplementary Information

**Additional file 1.**

## Data Availability

The datasets generated and/or analyzed during the current study are not publicly available because data analyses for other study aims are ongoing. Please contact Dr. Sannisha Dale at sdale@med.miami.edu with queries.

## Abbreviations

FPI: Five Point Initiative
EHE: Ending the HIV Epidemic
PrEP: Pre-exposure prophylaxis
CBPR: Community-based participatory research
CDC: Centers for Disease Control and Prevention
HRSA: Health Resources and Services Administration
SAMHSA: Substance Abuse and Mental Health Services Administration
CHO: Community health organizations
PHQ-9: Patient Health Questionnaire
