# Supplementary material for: Five point initiative: a community-informed bundled implementation strategy to address HIV in Black communities
Source: BMC Public Health. 2023 Aug 25;23:1625. doi: 10.1186/s12889-023-16525-7 (PMC10463742; doi:10.1186/s12889-023-16525-7)
Supplement: Supplementary file 1 — Additional file 1. [file 12889_2023_16525_MOESM1_ESM.docx]

**APPENDIX**

***Note: This overall administered survey includes some items from established scales published by other authors and citations are provided next to those items.***

1. Please create a unique identifier for yourself: (your first and last initials and your birth year) For example: MD1234
2. Do you live in the greater Miami area (Miami-Dade County, Broward County)?
3. If yes: What is your zip code? ________
4. if no: *End survey*
5. Were you born in the United States?
6. Yes
7. No (If no: in what country were you born? __________________)
8. Which of the following languages do you speak? (Check all that apply)
9. English
10. Kreyol
11. Español
12. Other __________________________________
13. What is your age? _____ (in years) (*if <18,* *End Survey*)
14. What is your current gender identity?
15. Male
16. Female
17. Trans male/trans man
18. Trans female/ trans woman
19. Genderqueer/gender non-conforming/non-binary
20. Different identity (please specify: ____________)
21. What sex were you assigned at birth, on your original birth certificate?
22. Male
23. Female
24. Intersex
25. Which of the following commonly used terms best describes your sexual orientation?
26. Heterosexual ( I am a woman attracted to men or a man attracted to women)
27. Gay
28. Lesbian
29. Bisexual
30. Queer
31. Pansexual
32. Asexual
33. Unsure/Questioning/Exploring
34. Not listed (please specify: ____________)
35. Do you consider yourself to be:
36. Black or African-American
37. Asian
38. White (including White Hispanic/Latino)
39. Native Hawaiian or other Pacific Islander
40. Native American
41. Multi-racial/Mixed (please specify: ____________)
42. Different racial identity (please specify: ____________)
43. Do you consider yourself to be (check all that apply):
44. Haitian
45. Afro-Caribbean Black (not Haitian)
46. Hispanic or Latino
47. Not Hispanic, Latino, Haitian, or Afro-Caribbean Black
48. Please indicate your highest or current education level:
49. Eighth grade or lower
50. Some high school
51. High school graduate or GED
52. Some college
53. College graduate
54. Some graduate school
55. Graduate school degree
56. I choose not to answer
57. Which of the following best describes your current housing arrangement? (Choose one)
58. Renting home or apartment
59. Living in home or apartment owned by you or someone else in household
60. Residential drug, alcohol or other treatment facility
61. Publicly subsidized housing (like Section 8)
62. A friend or relative's home/apartment (pays little or no rent)
63. Temporary/transitional housing (e.g., hotel, AIDS specific housing, Sober Living)
64. Homeless: sleeping in a shelter
65. Homeless: sleeping on the street, beach, car, etc.
66. Other Please Specify:____________________
67. I choose not to answer
68. What was your total household income, before taxes and other deductions, during the past 12 months?
69. Less than $5,000
70. $5,000 through $11,999
71. $12,000 through $15,999
72. $16,000 through $24,999
73. $25,000 through $34,999
74. $35,000 through $49,999
75. $50,000 and greater
76. Don’t Know
77. Refuse to Answer
78. Please check all that apply to you:
79. Full time work
80. Part time work
81. Full time or part time in school
82. Neither in work nor in school
83. On disability
84. Other
85. Please Specify: _____________________
86. I choose not to answer
87. In the past 12 months…I have been worried about whether our food run out before we got money to buy more. ^1^
88. Often
89. Sometimes
90. Never
91. In the past 12 months, the food I bought just didn’t last and I didn’t have money to get more. ^1^
92. Often
93. Sometimes
94. Never
95. Have you ever been incarcerated (locked up)?
96. Yes
97. No

(If yes, please select all that apply)

- 1. time in jail
  2. time in prison

1. How many children do you have? _____________________

18a. (If they have children) In your personal experience, has finding quality, affordable childcare that’s convenient for your family been: ^2^

1. Very easy
2. Somewhat easy
3. Somewhat difficult
4. Very difficult
   - 1. I haven’t needed childcare

18b. How serious of a problem is finding quality, affordable child care in your area? ^2^

1. Very serious problem
2. Somewhat serious problem
3. Not too serious of a problem
4. Not an issue in my community
5. Don’t know enough about it to answer
6. Over the last 2 weeks, how often have you been bothered by any the following problems? ^3,4^

19a. Feeling nervous, anxious, or on edge.

1. Not at all
2. Several Days
3. More than half the days
4. Nearly everyday

19b. Not being able to stop or control worrying.

1. Not at all
2. Several Days
3. More than half the days
4. Nearly everyday

19c. Little interest or pleasure in doing things.

1. Not at all
2. Several Days
3. More than half the days
4. Nearly everyday

19d. Feeing down, depressed, or hopeless.

1. Not at all
2. Several Days
3. More than half the days
4. Nearly everyday
5. I have high self-esteem ^5^

1 2 3 4 5

Not very true of me Very true of me

1. 1-Not very true of me
2. 2
3. 3
4. 4
5. 5-Very true of me
6. Have you ever experienced or witnessed or had to deal with a traumatic or violent event, that included actual or threatened death or serious injury or sexual violence to you or someone else, also any experience of rape, physical assault, car accident, community violence, sudden accidental death, serious accident, fire or explosion, being held captive, exposure to toxic substances, natural disaster, etc.? ^6^
7. Yes
8. No
9. In general, would you say your health is...?
10. Poor
11. Fair
12. Good
13. Very Good
14. Excellent
15. Do you have a primary care doctor/provider?
16. Yes
17. No
18. What is your HIV status?
19. HIV-positive, detectable viral load
20. HIV-positive, undetectable viral load
21. HIV-positive, I don’t know my viral load
22. HIV-negative
23. I don’t know
24. In your day-to-day life how often have any of the following things happened to you? ^7^

Answer choices:

1. You are treated with less courtesy or respect than other people.
2. You receive poorer service than other people at restaurants or stores.
3. People act as if they think you are not smart.
4. People act as if they are afraid of you.
5. You are threatened or harassed.
6. Almost everyday
7. At least once a week
8. A few times a month
9. A few times a year
10. Less than once a year
11. Never

***If the answer is “A few times a year” or more frequently to at least one question. What do you think is the main reason for these experiences? (Check all that apply).

1. Your Gender (being a woman or a man)
2. Your Gender Identity (e.g., Trans identified, gender nonconforming)
3. Your Race or Ethnicity (Black, Latinx, etc.)
4. Living with HIV
5. Your Sexual Orientation (LGBTQ)

The following questions ask about your opinions about medical care. Please use the following scale: strongly disagree, slightly disagree, neither agree nor disagree/unsure, slightly agree, or strongly agree. ^8^

1. Patients have sometimes been deceived or misled at hospitals. (Choose one)
2. Strongly Disagree
3. Slightly Disagree
4. Neither Agree Nor Disagree/Unsure
5. Slightly Agree
6. Strongly Agree
7. Hospitals often want to know more about your personal affairs or business than they really need to know. (Choose one)
8. Strongly Disagree
9. Slightly Disagree
10. Neither Agree Nor Disagree/Unsure
11. Slightly Agree
12. Strongly Agree
13. Hospitals have sometimes done harmful experiments on patients without their knowledge. (Choose one)
14. Strongly Disagree
15. Slightly Disagree
16. Neither Agree Nor Disagree/Unsure
17. Slightly Agree
18. Strongly Agree
19. Rich patients receive better care at hospitals than poor patients do. (Choose one)
20. Strongly Disagree
21. Slightly Disagree
22. Neither Agree Nor Disagree/Unsure
23. Slightly Agree
24. Strongly Agree
25. Male patients receive better care at hospitals than female patients do. (Choose one)
26. Strongly Disagree
27. Slightly Disagree
28. Neither Agree Nor Disagree/Unsure
29. Slightly Agree
30. Strongly Agree
31. Have you ever heard of PrEP (Pre-exposure Prophylaxis)? Yes/NO
32. (If no) Pre-exposure prophylaxis (PrEP) is a pill that a woman or man can take once a day to prevent getting HIV. PrEP is highly effective for preventing HIV, when taken every day.
33. Have you ever spoken to a healthcare provider about getting PrEP?
34. Yes
35. No
36. Are you currently prescribed:
37. *if HIV positive:* anti-retrovirals (HIV medication)?
    1. Yes
    2. No
38. *if HIV-negative or unknown:* pre-exposure prophylaxis (PrEP)?
    1. Yes
    2. No

If no:


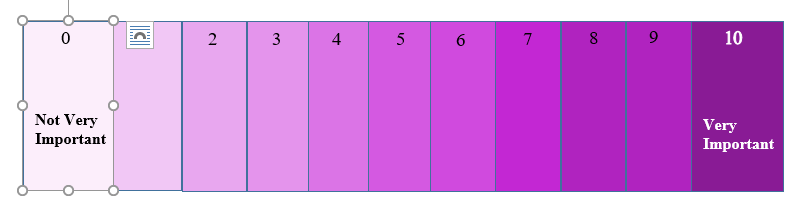


a. On a scale from 0-10, how important is it to you to start PrEP? ^9^


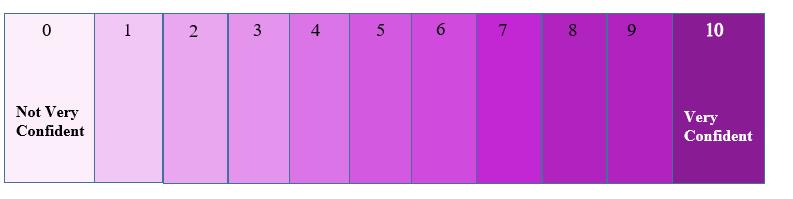


b. On a scale from 0-10, how confident are you that you will start using PrEP? ^9^

b. *If yes:* In the last 4 weeks, how good a job did you do at taking [*your medicine/PrEP*] in the way you were supposed to? ^10^

1. Poor
2. Fair
3. Good
4. Very Good
5. Excellent
6. Please select which of the following have made it difficult for you to receive the healthcare services you need. (Answer choices: Yes or No) ^11^
7. Long distances to medical facilities and personnel
8. Lack of transportation
9. Providers who do not speak your language
10. Lack of health care professionals who are adequately trained and knowledgeable
11. Shortage of psychologists, social workers, and mental health counsellors to help address mental health issues
12. Personal financial resources
13. Lack of adequate and affordable housing
14. Community residents’ stigma against persons living with HIV/AIDS
15. When was the last time you were tested for HIV?
16. In the last 3 months
17. 3 to 6 months ago
18. 6 to 12 months ago
19. Over 1 year ago
20. I can’t remember when
21. I have never been tested for HIV
22. Have you ever spoken to a doctor about HIV (for example, regarding testing, protection against HIV, treatment for HIV, etc.)?
23. Yes
24. No
25. Have you had any sex without a condom in the past 3-months?
26. Yes, one or two times
27. Yes, 3 to 5 times
28. Yes, more than 5 times
29. No
30. How many times in the past year have you had four or more drinks in a day? ^12^
31. Never
32. Less than once a month
33. A few times per month
34. Few times a week
35. More than 3x a week
36. How many times in the past year have you used a drug or used a prescription medication for non-medical reasons? ^13^
37. Never
38. Less than once a month
39. A few times per month
40. A few times per week
41. More than 3x per week

If answered b-e then

39a. What types of drugs did you use? (check all that apply)

1. Marijuana
2. Stimulants (e.g., meth, cocaine, crack)
3. Heroin
4. Club drugs/Hallucinogens (e.g., MDMA, Molly, ecstasy, LSD)
5. Tranquilizers (e.g., GHB, roofies)
6. Non-medical use of prescription medications (e.g., OxyContin, Vicodin, Fentanyl, Xanax, Valium, Ambien, Ritalin, Adderall)
7. How did you hear about today’s event? ________________________
   1. Friend or Family
   2. Word of mouth
   3. Flyer
   4. Event venue
8. Which parts of today’s event did you participate in?
   1. Completed survey
   2. Free HIV testing
   3. Free condoms
   4. Other ____________________________________
9. How satisfied were you with today’s event?
   1. Very satisfied
   2. Satisfied
   3. Neither satisfied nor unsatisfied
   4. Unsatisfied
   5. Very unsatisfied
10. I would participate in another community event like this one.
11. Strongly Agree
12. Agree
13. Neutral
14. Disagree
15. Strongly Disagree
16. Which of the following should we include in future events? (Check all that apply)
17. Tablets to conduct surveys
18. Vouchers
19. Host the event at similar locations (hairdressers, barbershops, corner stores, laundromats, mechanics)
20. Other _____________________
21. What are your thoughts on today’s event? ________________________________
22. If we were to do a research study in this location, how much time would you be willing to spend doing that study/intervention? (Number of minutes) _________________________
23. Less than 15 minutes
24. 15 to 30 minutes
25. 30 to 45 minutes
26. 45 minutes or above
27. What kinds of research study activities would you be willing to do in this location in exchange for voucher? (Yes or no for each item)
28. Talk to someone one on one
29. Watch videos
30. Complete surveys on tablets
31. Watch a speaker give a group presentation
32. Get tested for HIV and other STIs
33. Get a prescription for medication to prevent HIV or treat HIV

**REFERENCES**

1. *USDA ERS - Food Security in the U.S.*; 2022. Accessed February 24, 2022.

https://www.ers.usda.gov/topics/food-nutrition-assistance/food-security-in-the-u

s/survey-tools/#youth

2. Agne K, Halpin J, Omero M. *Affordable Child Care and Early Learning for All Families:*

*A National Public Opinion Study*.; 2022. Accessed February 24, 2022.

https://www.ers.usda.gov/topics/food-nutrition-assistance/food-security-in-the-u-s/

3. Kroenke K, Spitzer RL, Williams JB. The PHQ-9: validity of a brief depression severity

measure. *J Gen Intern Med*. 2001;16(9):606-613. doi:10.1046/j.1525

1497.2001.016009606.x

4. Williams JW, Mulrow CD, Kroenke K, et al. Case-finding for depression in primary care:

a randomized trial. *Am J Med*. 1999;106(1):36-43. doi:10.1016/s0002-9343(98)00371-4

5. Robins RW, Hendin HM, Trzesniewski KH. Measuring global self-esteem: Construct

validation of a single-item measure and the Rosenberg Self-Esteem Scale. *Pers Soc*

*Psychol Bull*. 2001;27:151-161. doi:10.1177/0146167201272002

6. *Diagnostic and Statistical Manual of Mental Disorders: DSM-5^TM^, 5th Ed*. American

Psychiatric Publishing, Inc.; 2013:xliv, 947. doi:10.1176/appi.books.9780890425596

7. Williams DR, Yan Yu null, Jackson JS, Anderson NB. Racial Differences in Physical

and Mental Health: Socio-economic Status, Stress and Discrimination. *J Health Psychol*.

1997;2(3):335-351. doi:10.1177/135910539700200305

8. LaVeist TA, Nickerson KJ, Bowie JV. Attitudes about racism, medical mistrust, and

satisfaction with care among African American and white cardiac patients. *Med Care Res*

*Rev MCRR*. 2000;57 Suppl 1:146-161. doi:10.1177/1077558700057001S07

9. Dale SK. Using Motivational Interviewing to Increase PrEP Uptake Among Black

Women at Risk for HIV: an Open Pilot Trial of MI-PrEP. *J Racial Ethn Health*

*Disparities*. 2020;7(5):913-927. doi:10.1007/s40615-020-00715-9

10. Lu M, Safren SA, Skolnik PR, et al. Optimal recall period and response task for self

reported HIV medication adherence. *AIDS Behav*. 2008;12(1):86-94.

doi:10.1007/s10461-007-9261-4

11. Heckman TG, Somlai AM, Peters J, et al. Barriers to care among persons living with

HIV/AIDS in urban and rural areas. *AIDS Care*. 1998;10(3):365-375.

doi:10.1080/713612410

12. Smith PC, Schmidt SM, Allensworth-Davies D, Saitz R. Primary Care Validation of a

Single-Question Alcohol Screening Test. *J Gen Intern Med*. 2009;24(7):783-788.

doi:10.1007/s11606-009-0928-6

13. Smith PC, Schmidt SM, Allensworth-Davies D, Saitz R. A single-question screening test

for drug use in primary care. *Arch Intern Med*. 2010;170(13):1155-1160.

doi:10.1001/archinternmed.2010.140
